# Post-diagnosis cannabis use and its association with health status and quality of life in cancer survivors

**DOI:** 10.1007/s00520-026-11185-w

**Published:** 2026-09-04

**Authors:** Nasim Kasiri, Corinne McDaniels-Davidson, Neal Doran, Sandip P. Patel, David R. Strong, Humberto Parada

**Affiliations:** 1 Division of Epidemiology and Biostatistics, School of Public Health, San Diego State University, San Diego, CA 92182 USA; 2 Division of Health Promotion and Behavioral Science, School of Public Health, San Diego State University, San Diego, CA 92182 USA; 3 UC San Diego Health Moores Cancer Center, La Jolla, CA 92037 USA; 4 Department of Radiation Medicine and Applied Sciences, School of Medicine, University of California San Diego, La Jolla, CA 92093 USA; 5 Department of Psychiatry, School of Medicine, University of California, San Diego, La Jolla, CA 92093 USA; 6 Psychology Service, VA San Diego Healthcare System, San Diego, CA 92161 USA; 7 Herbert Wertheim School of Public Health and Human Longevity Science, University of California, San Diego, La Jolla, CA 92093 USA

**Keywords:** Cannabis use, Cancer survivors, Health status, Physical health, Mental health, Quality of life

## Abstract

**Purpose:**

To examine whether measures of health status and quality of life (QoL) were associated with cannabis use after cancer diagnosis.

**Methods:**

Individuals who received care at an NCI-designated cancer center from 2018 to 2019 (*n* = 5,901) were invited to complete an online survey in 2022, of which 954 responded. We included 652 respondents in this study, which were weighted to represent the cancer center patient population [58.6% non-Hispanic White; 58.5% female; 51.8% ≥ 65 years]. We examined three self-rated exposures including physical health, mental health, and QoL each assessed using a single survey item with response options ranging from poor to excellent and dichotomized as ‘*Average or Below Average*’ or ‘*Above Average’* (referent). Post-diagnosis cannabis use was self-reported as ‘*Yes*’ or ‘*No’* (referent). Survey-weighted logistic regression was used to estimate adjusted odds ratios (ORs) and 95% confidence intervals (CIs), and to explore whether these associations differed by cancer stage, treatment status, or pre-diagnosis cannabis use.

**Results:**

Most reported above average status for mental health (67.2%) and QoL (64.2%), and almost half (47.1%) reported above average physical health. Odds of post-diagnosis cannabis use were significantly higher among survivors with average or below average mental health (OR = 1.88; 95%CI = 1.13–3.11). Associations were positive but not statistically significant for physical health (OR = 1.38; 95%CI = 0.85–2.24) and QoL (OR = 1.24; 95%CI = 0.75–2.05). These associations did not significantly differ by cancer stage, treatment status, or pre-diagnosis cannabis use.

**Conclusion:**

Cancer survivors reporting average or below average health status or QoL had higher odds of cannabis use.

## Introduction

Advancements in early cancer detection and treatment, along with a growing and aging population, have contributed to a steady rise in the overall number of cancer survivors. As of 2025, there were an estimated 18.6 million cancer survivors living in the United States (US) [1]. As cancer is increasingly managed as a chronic condition rather than a terminal illness, attention has shifted toward improving the long-term health and well-being of cancer survivors [1–3]. While many survivors return to normal functioning and live relatively symptom-free, a substantial proportion experience persistent or late-emerging physical and psychological effects of cancer and its treatments [2]. These effects, including pain and emotional distress, can negatively affect daily functioning and overall quality of life (QoL) [3, 4]. As a result, many survivors actively pursue supportive therapies, complementary approaches, and self-management strategies to improve their health and well-being following cancer diagnosis and treatment [5, 6].

One such complementary approach that has gained significant attention among cancer survivors is cannabis use. In the US, cannabis use has become increasingly prevalent among adults, especially those with chronic health conditions [7–9]. This trend has coincided with evolving legal landscapes, more permissive social attitudes towards cannabis, and increasing perceptions of its safety [7–9]. Medical cannabis is now legal in most US states, with approved indications often including cancer- or cancer treatment-related symptoms including pain, nausea, insomnia, appetite loss, and mood disorders (e.g., anxiety and depression) [10, 11]. In our previous work, we reported that over 80% of surveyed cancer survivors perceived benefits from cannabis use, with most citing pain relief, reduced stress, anxiety, or depression, and improved management of treatment-related side effects as primary benefits of using cannabis [12]. However, the evidence regarding cannabis efficacy for cancer-related symptom management remains limited. The American Society of Clinical Oncology guidelines on cannabis and cannabinoids in adult cancer care noted insufficient evidence to recommend for or against cannabis use for most cancer-related symptoms, which highlights a gap between perceived benefits and established evidence [10].

Although previous studies have described the prevalence of cannabis use and reasons for use among individuals with cancer, relatively few studies have explored how such use relates to broader indicators of survivorship health such as physical functioning, mental well-being, or QoL. Most existing studies have framed cannabis use primarily as a palliative tool emphasizing its role in addressing unmet symptom needs, particularly among individuals with high symptom burden or advanced disease [13–16]. Building on this perspective, survivors experiencing ongoing physical or psychological challenges may be more likely to use cannabis post-diagnosis to manage symptoms, improve comfort, or support daily functioning. This pattern of use may reflect a more reactive approach, in which individuals turn to cannabis to cope with lower health status or diminished QoL. As such, cannabis use in survivorship may be more prevalent among those reporting poorer health than among those with fewer ongoing issues or higher levels of well-being.

The primary aim of this study was to evaluate whether self-rated physical health, mental health, and overall QoL were associated with cannabis use following a cancer diagnosis. We hypothesized that cancer survivors who reported average or below average health status across physical, mental, and QoL domains would have higher odds of using cannabis after diagnosis. Additionally, we examined whether these associations varied by cancer stage at diagnosis as cancer severity may influence the likelihood of post-diagnosis cannabis use among survivors, treatment status as survivors in active treatment may experience more treatment-related side-effects, and by pre-diagnosis cannabis use as those who have previous experience using cannabis may be more comfortable acquiring or discussing cannabis use compared to never-users.

## Methods

### Study design and data collection

Data were collected by electronic survey administered using Qualtrics between March and June 2022 at the University of California San Diego Health Moores Cancer Center (MCC), a National Cancer Institute (NCI)-designated Comprehensive Cancer Center in Southern California. Individuals who received care at MCC between January 2018 and December 2019 and who had valid email addresses were invited to complete the online survey. The main purpose of the survey was to assess cannabis-related knowledge, attitudes, and behaviors among cancer survivors, along with their individual cancer experiences and related symptoms, as previously described [12, 17]. Study materials were provided in both English and Spanish, and participants received a $10 gift card as an incentive for completing the survey. To ensure privacy, survey responses were not linked to any identifying information; however, IP addresses were recorded to guarantee a single submission per invitation. The study underwent review and approval by the institution’s affiliated Human Research Protections Program and the Protocol Review and Monitoring Committee. Informed consent was obtained from all participants prior to survey completion.

### Study population

A total of 5,901 individuals were eligible and invited to participate in the study, of which 954 (16.2%) responded. A third-party company weighted the final analytic responses to ensure that the sample was representative of the MCC patient population. A total of 268 respondents were excluded from analytic estimates using a domain subpopulation statement, as described in the Statistical Analysis section of the Methods. Respondents were excluded if they reported a stage 0 cancer diagnosis (*n* = 134). Additionally, respondents with missing data on cancer stage at diagnosis (*n* = 73), post-cancer diagnosis cannabis use (*n* = 40), or self-rated physical health, mental health, and QoL (*n* = 34) were excluded. The final analytic sample consisted of 652 respondents, resulting in a weighted sample size of 4,620.

### Post-cancer diagnosis cannabis use

The primary outcome of interest, post-cancer diagnosis cannabis use, was assessed by a survey question that asked, “*Have you used cannabis at any time since your cancer diagnosis?*” Participants could respond with either ‘*Yes*’ or ‘*No*’ (referent). Cannabis use comprised both tetrahydrocannabinol (THC)-containing and cannabidiol (CBD)-containing products including marijuana, cannabis concentrates, CBD-only products, pharmaceutical or prescription cannabinoids, edibles, lotions, ointments containing cannabis, or other products made with cannabis. The survey also collected information on cannabis use prior to cancer diagnosis and whether participants had considered using cannabis following their cancer diagnosis, which were used to further characterize cannabis use patterns among cancer survivors.

### Self-rated physical health, mental health, and overall quality of life

We examined three self-rated exposures including physical health, mental health, and QoL. The survey questions asked, “*In general, how would you describe your…Physical health? Mental health? Overall quality of life?*” Response options ranged from poor to excellent and were dichotomized as ‘*Average or Below Average*’ (i.e., good, fair, or poor) and ‘*Above Average*’ (very good or excellent [referent]) to ensure sufficient numbers of observations in each category.

### Covariates

All covariates were assessed by participant self-report. We considered the following covariates as potential confounders of the associations between physical health, mental health, and QoL and post-cancer diagnosis cannabis use: age range in years at the time the survey was completed (25–34, 35–44, 45–54, 55–64, 65–74, 75–84, 85–94, or ≥ 95 years); sex (male or female); race/ethnicity (non-Hispanic White, non-Hispanic Black, Asian, Hispanic/Latino or other); education level (≤ high school or post-high school training, some college, college graduate, or postgraduate); marital status (married, separated, or single); occupation status (employed, unemployed, retired, disabled, or other); cancer type by body location and system (breast, digestive/gastrointestinal, endocrine/neuroendocrine, genitourinary, gynecologic, head and neck, hematologic/blood, neurologic, respiratory, skin, or other as defined by NCI [18]); and tobacco use (current smoker, former smoker, or never smoker based on lifetime use of ≥ 100 cigarettes and frequency of current cigarette use [e.g., every day, some days, not at all]). We used directed acyclic graphs [19] to identify potential confounders and mediators of the associations between using cannabis after a cancer diagnosis and the health status indicators. We considered the following covariates as potential modifiers of the associations between physical health, mental health, and QoL and post-cancer diagnosis cannabis use: cancer stage (non-advanced [stages I/II] and advanced [stages III/IV]); treatment status (newly diagnosed or undergoing treatment, and post-treatment cancer survivor); and pre-cancer diagnosis cannabis use (ever used and never used).

### Statistical analyses

Survey-weighted multivariable logistic regression was used to estimate odds ratios (ORs) and 95% confidence intervals (CIs) for the associations between post-cancer diagnosis cannabis use and physical health, mental health, and QoL. Each exposure was assessed in separate models given the moderate-to-strong Pearson correlations observed between the three continuous health status measures (*r*_physical health-mental health_ = 0.46; *r*_physical health-QoL_ = 0.54; and *r*_mental health-QoL_ = 0.61). All logistic regression models adjusted for age, sex at birth, race/ethnicity, cancer type by body location and system, cancer stage at diagnosis, treatment status, and pre-cancer diagnosis cannabis use. We examined effect measure modification by stratifying the logistic regression models by cancer stage at diagnosis, treatment status, and pre-cancer diagnosis cannabis use, and by including in fully adjusted models multiplicative interaction terms between effect modifiers and each of the health status measures. Domain analyses were used to obtain valid survey-weighted estimates while preserving the original sampling design and variance estimation when analyses were restricted to the analytical subset. Hypothesis tests were two-sided with α = 0.05, and all analyses were conducted using SAS version 9.4 (SAS Institute Inc., Cary, NC USA).

## Results

The weighted demographic characteristics of the study population are summarized in Table 1. Approximately half of the survivors (51.8%) were aged 65 years or older, with 33.2% falling within the 65–74 age group. The majority of the population was female (58.5%) and self-identified as non-Hispanic White (58.5%). Roughly 16% self-identified as Hispanic or Latino. Nearly half (47.8%) were retired, 68.7% were married, and 64.9% had completed a college degree or held a postgraduate degree. Current cigarette smoking was reported by 3.6% of the population.

**Table 1:** Descriptive statistics of the adult cancer survivors from the NCI-designated Comprehensive Cancer Center in Southern California (*n* = 4,620)

Characteristic	*n*_weighted_	%_weighted_
Age, years		
25–34	144	3.4
35–44	369	8.7
45–54	559	13.2
55–64	977	23.0
65–74	1,412	33.2
75–85	680	16.0
85–94	103	2.4
≥ 95	8	0.2
Sex		
Female	1,602	58.5
Male	1,846	41.5
Race/Ethnicity		
Non-Hispanic White	2,706	58.5
Non-Hispanic Black	192	4.2
Asian	479	10.4
Hispanic	730	15.8
Other	514	11.1
Education		
≤ High School	793	17.9
Some College	767	17.3
College Graduate	1,426	32.1
Post-Graduate	1,454	32.8
Cigarette Smoking Status		
Never smoker	2,917	65.1
Former smoker	1,404	31.3
Current smoker	162	3.6
Marital Status		
Married	2,986	68.7
Separated	936	21.5
Single	422	9.7
Occupational Status		
Employed	1,920	33.2
Unemployed	376	6.5
Retired	2,764	47.8
Disabled	343	5.9
Other	379	6.6

The weighted frequencies of cancer- and cannabis-specific variables for the study population are presented in Table 2. More than half were diagnosed with non-advanced stages of cancer; stage I (37.1%), stage II (24.0%), stage III (21.5%), and stage IV (17.4%). Over three-quarters (76.1%) identified as post-treatment cancer survivors, with 21.4% undergoing active treatment at the time of survey completion. The most commonly reported cancer types were breast (30.1%) followed by genitourinary system (18.0%) cancers. Approximately half (51.9%) reported cannabis use prior to their cancer diagnosis, while 32.0% reported post-diagnosis use, and 14.6% reported considering using cannabis. About two-thirds rated their mental health (67.2%) and QoL (64.2%) as above average, while almost half (47.1%) rated their physical health as above average.

**Table 2:** Cancer- and cannabis-specific statistics of the adult cancer survivors from the National Cancer Institute-designated Comprehensive Cancer Center in Southern California (*n* = 4,620)

Characteristic	*n*_weighted_	%_weighted_
Cancer Stage at Diagnosis		
I	1,714	37.1
II	1,110	24.0
III	992	21.5
IV	804	17.4
Cancer Treatment Status		
Newly Diagnosed	111	2.5
Undergoing Treatment	938	21.4
Post-Treatment Cancer Survivor	3,335	76.1
Cancer Type by Body Location and System		
Breast	1,384	30.1
Digestive/Gastrointestinal	490	10.6
Endocrine and Neuroendocrine	224	4.9
Genitourinary	829	18
Gynecologic	396	8.6
Head and Neck	218	4.7
Hematologic/Blood	409	8.9
Neurologic	114	2.5
Musculoskeletal	60	1.3
Respiratory/Thoracic	266	5.8
Skin	188	4.1
Other	29	0.6
Pre-Cancer Diagnosis Ever Cannabis Use		
No	2,212	48.1
Yes	2,384	51.9
Post-Cancer Diagnosis Ever Cannabis Use		
No	3,141	68.0
Yes	1,479	32.0
Post-Cancer Diagnosis Consideration of Cannabis Use		
No	2,666	85.4
Yes	456	14.6
Self-Rated Physical Health Status		
Average or Below Average	2,443	52.9
Above Average	2,177	47.1
Self-Rated Mental Health Status		
Average or Below Average	1,517	32.8
Above Average	3,103	67.2
Self-Rated Overall Quality of Life		
Average or Below Average	1,652	35.8
Above Average	2,968	64.2

In Table 3, we report the associations between physical health, mental health, and overall QoL and post-diagnosis cannabis use. Those who rated their mental health as average or below average had significantly higher odds of post-diagnosis cannabis use compared to those with above average mental health (OR = 1.88; 95%CI = 1.13–3.11). Associations were positive but not statistically significant for physical health (OR = 1.38; 95%CI = 0.85–2.24) and QoL (OR = 1.24; 95%CI = 0.75–2.05).

**Table 3:** Odds ratios (ORs) and 95% confidence intervals (CIs) for the associations between physical health, mental health, and overall quality of life and post-diagnosis cannabis use

Health status	OR (95% CI)^a^
Physical Health	
Average or Below Average	1.38 (0.85–2.24)
Above Average	1.00 (Referent)
Mental Health	
Average or Below Average	1.88 (1.13–3.11)
Above Average	1.00 (Referent)
Overall Quality of Life	
Average or Below Average	1.24 (0.75–2.05)
Above Average	1.00 (Referent)

^a^Adjusted for age, sex, race/ethnicity, cancer type by body location and system, cancer stage at diagnosis, treatment status, and pre-cancer diagnosis use of cannabis using survey-weighted multivariable logistic regression models

In Table 4, we report the associations between physical health, mental health, and overall QoL and post-diagnosis cannabis use by cancer stage at diagnosis, treatment status, and pre-cancer diagnosis cannabis use. None of the interactions were statistically significant (*P*_Interactions_ ≥ 0.12). However, in stratified analyses, average or below average mental health was associated with higher odds of post-diagnosis cannabis use among survivors diagnosed with non-advanced cancer (OR = 2.15; 95%CI = 1.12–4.12), survivors who were post-cancer treatment (OR = 2.01; 95%CI = 1.13–3.56), and survivors who reported never using cannabis prior to diagnosis (OR = 4.26; 95%CI = 1.34–13.58). No other stratified associations were statistically significant.

**Table 4:** Odds ratios (ORs) and 95% confidence intervals (CIs) for the associations between physical health, mental health, and overall quality of life and post-diagnosis cannabis use stratified by cancer stage at diagnosis, treatment status, and pre-diagnosis use of cannabis

CANCER STAGE AT DIAGNOSIS	Non-Advanced Cancer Stage (Stages I/II)	Advanced Cancer Stage (Stages III/IV)	*P*_Interaction_^b^
	OR (95% CI)^a^	OR (95% CI)^a^	
Physical Health			0.38
Average or Below Average	1.77 (0.94–3.32)	0.86 (0.34–2.19)	
Above Average	1.00 (Referent)	1.00 (Referent)	
Mental Health			0.66
Average or Below Average	2.15 (1.12–4.12)	1.83 (0.76–4.41)	
Above Average	1.00 (Referent)	1.00 (Referent)	
Overall Quality of Life			0.71
Average or Below Average	1.41 (0.76–2.62)	1.06 (0.41–2.73)	
Above Average	1.00 (Referent)	1.00 (Referent)	
TREATMENT STATUS	Newly Diagnosed or Undergoing Treatment	Post-Cancer Treatment	*P*_Interaction_^b^
	OR (95% CI)^a^	OR (95% CI)^a^	
Physical Health			0.12
Average or Below Average	2.92 (0.72–11.82)	1.19 (0.69–2.05)	
Above Average	1.00 (Referent)	1.00 (Referent)	
Mental Health			0.77
Average or Below Average	1.18 (0.34–4.03)	2.01 (1.13–3.56)	
Above Average	1.00 (Referent)	1.00 (Referent)	
Overall Quality of Life			0.31
Average or Below Average	2.49 (0.70–8.79)	1.17 (0.66–2.07)	
Above Average	1.00 (Referent)	1.00 (Referent)	
PRE-CANCER DIAGNOSIS CANNABIS USE	Ever Used	Never Used	*P*_Interaction_^b^
	OR (95% CI)^a^	OR (95% CI)^a^	
Physical Health			0.29
Average or Below Average	1.07 (0.60–1.92)	2.87 (0.84–9.75)	
Above Average	1.00 (Referent)	1.00 (Referent)	
Mental Health			0.62
Average or Below Average	1.53 (0.87–2.68)	4.26 (1.34–13.58)	
Above Average	1.00 (Referent)	1.00 (Referent)	
Overall Quality of Life			0.58
Average or Below Average	1.13 (0.64–2.00)	1.87 (0.60–5.80)	
Above Average	1.00 (Referent)	1.00 (Referent)	

^a^Adjusted for age, sex, race/ethnicity, cancer type by body location and system, cancer stage at diagnosis, treatment status, and pre-cancer diagnosis use of cannabis, as appropriate, using survey-weighted multivariable logistic regression models

^b^*P*-values for the interaction terms between each self-rated health measure and cancer stage at diagnosis, treatment status, and pre-cancer diagnosis cannabis use

## Discussion

We examined whether self-rated physical health, mental health, and overall QoL were associated with cannabis use following a cancer diagnosis and whether these associations varied by cancer stage at diagnosis, treatment status, and pre-diagnosis cannabis use. Consistent with our hypothesis, survivors reporting average or below average physical health, mental health, and QoL were more likely to use cannabis after their diagnosis; however, only the association between mental health and post-diagnosis cannabis use was statistically significant. Additionally, while none of the interactions were statistically significant, average or below average mental health was significantly associated with higher odds of post-diagnosis cannabis use among survivors diagnosed with non-advanced cancer, post-cancer treatment survivors, and those who had never used cannabis prior to diagnosis. Of note, these findings should be interpreted with caution as estimates were imprecise.

Although limited, most previous studies have reported that cancer survivors who use cannabis tend to have lower QoL compared to those who do not use cannabis [20–23]. These studies, conducted primarily among colorectal cancer survivors and oncology patients, assessed QoL using validated multi-item instruments including the 30-item European Organization for Research and Treatment of Cancer Quality of Life Questionnaire-Core 30 [20, 22], the Functional Assessment of Cancer Therapy-Colon [21], the Edmonton Symptom Assessment Score (ESAS) [23]. Across these studies, cannabis use was generally associated with poorer QoL or symptom outcomes, although Nathan et al. (2023) found that ESAS scores remained stable following cannabis initiation among older adults receiving medical cannabis for cancer-related symptoms [23]. These previous studies used comprehensive methodological approaches to evaluate cancer-related QoL among cannabis users and their findings were largely consistent with ours. Despite our reliance on single-item measures of health status and QoL, this consistency suggests that brief self-rated assessments may capture meaningful differences in health status associated with post-diagnosis cannabis use.

While previous studies have associated cannabis use with lower overall QoL, they have generally treated QoL as a single composite construct and have not examined physical health and mental health as distinct health domains. One exception is Xu et al. [24], who used Behavioral Risk Factor Surveillance System data to assess physically and mentally unhealthy days among cancer survivors and found that frequent cannabis use was associated with poorer outcomes across both domains. Do et al. [25] evaluated physical and mental health status as predictors of cannabis use using nationally representative longitudinal data but found that mental health was a significant predictor only among individuals without a history of cancer. Our study builds on this work by examining self-rated physical health, mental health, and overall QoL as separate health domains in relation to post-diagnosis cannabis use specifically among cancer survivors. This approach showed that average or below average mental health was the only health domain significantly associated with post-diagnosis cannabis. Our examination of effect measure modification by cancer stage at diagnosis, treatment status, and pre-diagnosis cannabis use represents a novel contribution, as previous studies have not systematically evaluated whether these associations differ by these characteristics.

Cancer stage at diagnosis is a key clinical marker associated with increased disease severity, treatment intensity, and prognosis. We hypothesized that cancer survivors diagnosed at more advanced stages would face more intensive treatment, ongoing symptoms, and greater physical and psychological burden that may motivate cannabis use [26]. In our prior work, we found that survivors diagnosed at stages III/IV had 63% higher odds of considering cannabis use compared to those diagnosed at stages I/II [17]. However, in this study, we found little evidence of heterogeneity in the associations between health status and QoL and post-diagnosis cannabis use, although stratified analyses suggested potentially higher odds of post-diagnosis cannabis use among survivors with average or below average mental health who were post-cancer treatment or who had never used cannabis prior to diagnosis. We hypothesize that survivors who are in post-treatment phases may experience fear of recurrence, persistent symptoms, financial stress, and reduced clinical support and may therefore turn to cannabis for emotional regulation, sleep, or coping during this period. Our findings by pre-cancer diagnosis use of cannabis suggest that cancer diagnosis may serve as an initiation point for cannabis use among some previously cannabis-naïve survivors, potentially for symptom management; however, this result may also reflect broad measurement of lifetime pre-cancer diagnosis use, post-diagnosis cessation among former users, reporting error, or residual confounding. Altogether, these findings suggest that both the decision to use cannabis and its perceived benefits may be shaped by survivors’ symptom burden, current health status, and previous experience with cannabis.

Cannabis use after a cancer diagnosis may reflect a symptom-driven, reactive approach to managing ongoing physical and psychological challenges, particularly among survivors who are no longer in active treatment but continue to experience long-term effects of cancer or its treatment. In our previous study, we found that survivors who perceived any of 15 listed benefits of cannabis use had more than a five-fold increase in the odds of reporting post-diagnosis cannabis use, whereas perceiving any risks was associated with a 59% decrease in the odds of post-diagnosis cannabis use [12]. Rather than representing a proactive pursuit of wellness, this pattern of use appears more closely tied to efforts to relieve distress or discomfort. This aligns with frameworks such as Symptom Management Theory, which highlights the central role of symptom experience and relief-seeking in patient behavior [27, 28]. In this context, cancer survivors may turn to cannabis to manage cancer-related symptoms while also considering potential side effects and broader social perceptions of use [29]. The Self-Medication Hypothesis further supports this interpretation, proposing that individuals may use substances like cannabis to cope with persistent physical or psychological distress [30–32], which is supported by our findings which consistently highlight mental health as a correlate of cannabis use among cancer survivors and thus a potential target of intervention given that clinicians may not be aware of cannabis use behaviors. Continued research is needed to better understand the complex and multifaceted drivers of cannabis use among cancer survivors.

This study has several strengths that enhance the robustness and interpretability of the findings. This study is based on a large, weighted sample drawn from an NCI-designated Comprehensive Cancer Center, allowing for greater generalizability to more diverse cancer survivor populations within academic clinical settings. The single-institution design may minimize variability in cancer care delivery, potentially reducing confounding related to treatment access or quality of care. Cannabis accessibility in California, including San Diego, is shaped by state-level legalization of medical and recreational cannabis during the study period, which may influence patterns of use and limit generalizability to regions with different legal frameworks. However, several limitations should be noted. The cross-sectional study design precludes assessment of temporality and does not allow descriptions of the causal direction of observed associations between cannabis use and self-rated health status and QoL. Because cannabis use and health outcomes are assessed concurrently in cross-sectional studies, the directionality of these associations remains unclear and may reflect bidirectional relationships. All single-item measures of health status and cannabis use were subject to recall or social desirability bias. While multi-item QoL scales are valuable for clinical and psychometric applications, the use of single-item QoL measures is well supported in epidemiological research and has several advantages including being more time- and cost-effective, while being equally valid and reliable [33–38]. Cannabis use was not verified through medical records or biological measures, potentially resulting in misclassification. Additionally, THC and CBD were assessed together and are notably different compounds with distinct pharmacologic effects; combining them precludes examination of differential associations with health outcomes and may obscure potential variation in benefits or risks related to specific cannabinoid use. Other aspects of the patient’s cancer journey such as information on treatment modality, time since diagnosis, and recurrence history were not collected, but may be important to consider as these factors may provide insight into the differences in self-rated health status across survivors. Although survey weights were applied to improve representativeness, the response rate was modest, and selection bias remains a possibility. Finally, residual confounding from unmeasured variables like provider recommendations or concurrent use of other complementary therapies cannot be ruled out as these factors may influence the survivor’s health status.

Future research should aim to better understand the diversity of the survivorship experience and how cannabis use fits into this landscape. As survivorship experiences vary, important differences in health status may emerge based on a multifactorial combination of clinical characteristics, treatment history, and psychosocial or behavioral factors. Future work therefore must investigate how these factors influence cannabis use and whether survivors with different clinical histories turn to cannabis for distinct reasons. Doing so would help ensure more reliable comparisons across studies and support a clearer understanding of how cannabis use relates to survivors’ perceived well-being. Longitudinal studies in particular will help clarify the timing and persistence of cannabis use relative to changes in health status. Qualitative approaches will also be important for adding depth by capturing how survivors perceive cannabis as part of their broader coping or wellness strategies. Incorporating detailed measures of cannabis exposure such as product type, frequency, and route of administration, will be important for guiding clinical discussions and informing survivorship care planning.

## Conclusion

In this study, cancer survivors reporting poorer mental health had higher odds of post-diagnosis cannabis use, particularly among those who reported never using cannabis prior to diagnosis. Understanding how cannabis use varies across different survivorship experiences will be critical for developing patient-centered care that respects individual needs and values. As the role of cannabis in cancer care continues to grow, it is important that clinicians and researchers consider not only who is using cannabis in this context, but also who is not and why. Building a more robust evidence base can also help ensure that cannabis use can be thoughtfully and effectively integrated into survivorship care where appropriate, while also empowering survivors to make informed decisions about their health and well-being.

## Data Availability

The data that support the findings of this study are openly available through the UC San Diego Library Digital Collections at https://doi.org/10.6075/J0NP24MQ.
